# Leishmaniose cutanée érysipéloide: à propos d'une observation clinique

**DOI:** 10.11604/pamj.2015.21.54.5967

**Published:** 2015-05-25

**Authors:** Abdeslam El kartouti, Jalal Elbenaye, Mouhcine Miloudi

**Affiliations:** 1 Service de Pharmacie Hospitalière, 3ème Hôpital Militaire Laayoune, Université Sidi Mohamed Ben Abdallah, Faculté de Médecine et de Pharmacie de Fès, Fès Maroc; 2 Service de Dermatologie, 3ème Hôpital Militaire, Laayoune, Maroc; 3 Service de Biologie Médicale, 3ème Hôpital Militaire, Laayoune, Maroc

**Keywords:** Leishmaniose cutanée, forme érysipéloide, antimoniate de méglumine, cutaneous leishmaniasis, erysipeloid form, meglumine antimoniate

## Abstract

Les auteurs rapportent les caractéristiques épidémiologiques et cliniques de la forme érysipéloïde de leishmaniose cutanée ainsi que ses difficultés diagnostiques et thérapeutiques. Chez une patiente âgée de 44 ans, sans antécédents, a consulté pour une tuméfaction nasale inflammatoire évoluant depuis 4 mois. L'examen clinique a révélé un placard érythémateux, infiltré centrofaciale. Une antibiothérapie avec des soins locaux quotidiens n'ont pas entraîné une amélioration, voire l'extension de lésions sous anti-inflammatoire non stéroïdiens. Le diagnostic de leishmaniose cutanée a été confirmé par le frottis cutané. Un traitement par l'antimoniate de méglumine par voie intramusculaire a été instauré à la dose de 20 mg/kg par jour avec évolution favorable. La forme érysipéloïde de leishmaniose cutanée constitue une entité rare et inhabituelle entraînant souvent un retard diagnostique. Le diagnostic repose sur l'examen parasitologique direct, la recherche de l'ADN des leishmanies par PCR et sur l'examen histologique. Et il existe plusieurs options thérapeutiques. L’évolution est généralement favorable.

## Introduction

La leishmaniose cutanée (LC) représente un problème de Santé publique à l’échelle mondiale, est une maladie parasitaire très répandue, due à une piqûre infectante d'un insecte vecteur, le phlébotome. Elle est endémique dans plus de 70 pays avec une incidence annuelle estimée à 1500 000 cas [1–3]. Ces dernières années, il a été constaté, une augmentation du nombre de cas de cette maladie au Maroc [4]. En effet, en 2010, le nombre de cas déclarés de LC à Leishmania major était de 6444 contre 4402 en 2009 et 3431 en 2008 [5]. Nous rapportons un cas de LC érysipéloide faciale, forme rare, dans le but d’évoquer les particularités épidémiologiques, cliniques et thérapeutiques de cette forme.

## Patient et observation

**Figure 1:**
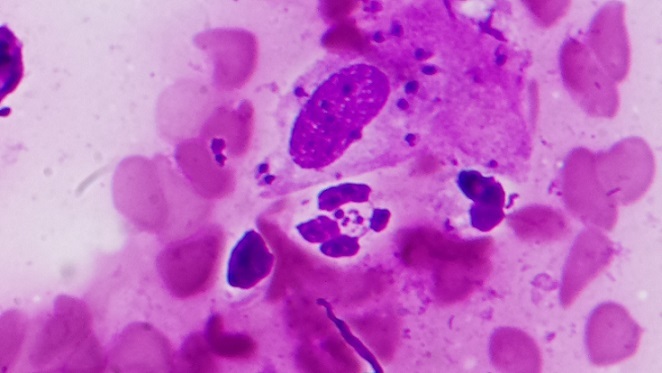
Frottis cutané montrant des amastigotes au sein de macrophages

**Figure 2:**
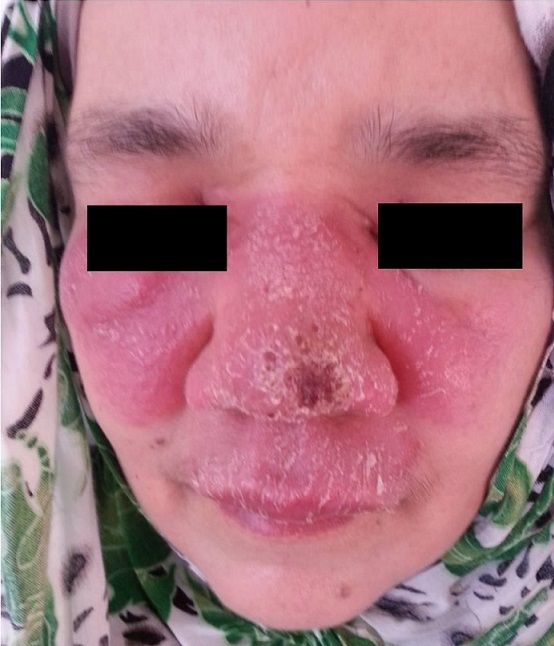
Placard érythémateux œdémateux, squameux et infiltré couvrant de façon symétrique la région centrofaciale

Il s'agit d'une femme de 44 ans, sans antécédents pathologiques particuliers, ménopausée depuis une année et demie, qui a consulté pour une tuméfaction nasale évoluant depuis 4 mois. L'examen clinique a révélé un placard érythémateux œdémateux et squameux en aile de papillon, couvrant de façon symétrique la région centrofaciale (nez et joues) (Figure 1) et évoquant un érysipèle du visage. L'examen objective des croutes sur la pointe du nez, révèle des lésions indolores et sans atteinte muqueuse sous jacente ni de signes systémiques. La patiente était apyrétique et tout le bilan biologique et inflammatoire était normal. La patiente rapporte qu'elle avait reçu plusieurs classes d'antibiotiques sans amélioration. Notre patiente rapporte l'extension de la lésion nasale initiale 2 jours après la prise des anti-inflammatoire non stéroïdiens (AINS). Devant ces éléments cliniques, plusieurs diagnostics sont évoqués dont la leishmaniose cutanée, laquelle a été confirmée par un frottis cutané en montrant des amastigotes de leishmanies au sein de macrophages (Figure 2). Le diagnostic par la recherche de l'ADN des leishmanies par PCR et sur l'examen histologique, n'a pas été fait pour notre cas par manque de moyens. Une cure systémique d'injections intramusculaires d'antimoniate de méglumine (Glucantime^®^) à la dose de 20 mg/kg/j d'antimoine sans dépasser 850 mg/j pendant 20j a été initiée avec une bonne tolérance et un suivi clinique et biologique satisfaisant. Ainsi une amélioration clinique nette est observée à après 2 semaines de traitement et une rémission complète est obtenue à partir d'un mois d'arrêt du traitement. Toutefois, la patiente garde un érythème rosé couperosique sans infiltration.

## Discussion

La leishmaniose cutanée (LC) représente au Maroc un problème majeur de Santé publique, elle est désormais une maladie à déclaration obligatoire depuis 1995. Une recrudescence des cas de LC a été constatée ces dernières années. Cette pathologie est due a trois espèces du genre Leishmania: L. major, L. tropica et L. infantum [4]. Il y avait un polymorphisme clinique des lésions avec prédominance de l'aspect ulcéro-croûteux observé dans 55,1% des cas. L'atteinte faciale était observée dans 20,4% des cas [6]. La guérison spontanée se fait en quelques mois, laissant une cicatrice claire ou pigmentée ainsi qu'une immunité durable. La LC dans sa forme érysipéloide, forme rare ayant été rapportée selon la littérature en Iran, au Pakistan, en Tunisie et en Turquie [7–11]. Cette forme diffère des autres par ses caractéristiques cliniques mais aussi par la population qu'elle touche préférentiellement [10]. Selon les données de la littérature, la fréquence de la LC érysipéloïde varie entre 0,05 et 3,2% [7, 9]. Cette forme touche préférentiellement les femmes âgées [10]. Elle se manifeste cliniquement par un placard érythémateux, infiltré et diffus de la face couvrant le nez et les deux joues [7], comme c'est le cas chez notre patiente. La cause de survenue de cette forme est inconnue. Parmi les hypothèses évoquées comme à l'origine de cette forme de LC, le déficit de la réponse immunitaire de l'hôte lié à la sénilité, un type particulier de parasite, les perturbations hormonales à la période de la ménopause, une altération de la qualité de la peau due au vieillissement. Notre patiente, bien qu'elle ne soit pas âgée, présente deux caractéristiques épidémiologiques en faveurs de cette forme particulière; elle est originaire d'une région endémique de leishmaniose où un chevauchement des aires de répartition existe entre L. infantum et L. tropica [4, 12] et femme en période de ménopause avec toutes les perturbations hormonales associées.

L'atteinte localisée au niveau du visage, a été à l'origine d'un retard diagnostique chez notre patiente, puisque ces lésions peuvent évoquer d'autres diagnostics tels qu'une infection cutanée bactérienne ou fongique, la syphilis, l'anthrax ou un eczéma [11]. Ainsi, notre patiente a bénéficié préalablement à notre consultation de la prescription de plusieurs classes d'antibiotiques et d'anti-inflammatoire non stéroïdiens, mais sans amélioration. Notre patiente a rapporté également l'extension de la lésion nasale après traitement par anti-inflammatoire. Le diagnostic de la leishmaniose cutanée a été évoqué par la suite, dont la confirmation a été réalisée par un frottis cutané ayant montré des amastigotes de leishmanies au sein de macrophages. Sur le plan thérapeutique, Il existe plusieurs options thérapeutiques telles que la cryothérapie, le traitement par la chaleur grâce à la radiofréquence, un traitement topique, les traitements oraux tel que le fluconazole, le métronidazole [13]. Notre patiente a reçu de l'antimoniate de méglumine (Glucantime^®^), qui reste le médicament le plus utilisé pour le traitement des LC. Ce traitement se fait par infiltrations locales lorsqu'il s'agit de lésions uniques en dehors du visage. Quand les lésions sont multiples ou lors d'atteinte faciale, le traitement se fait par injections intramusculaires. La forme érysipéloïde touchant le visage impose un traitement injectable. Chez notre patiente, le traitement a été initié par des injections intramusculaires d'antimoniate de méglumine (Glucantime^®^) à la dose de 20mg/kg/j d'antimoine sans dépasser 850mg/j pendant 20 jours avec une bonne tolérance et un suivi clinique et biologique satisfaisant. Ce médicament présente de nombreux effets indésirables tels que douleurs musculaires, atteinte rénale, toxicité hépatique et cardiaque [14]. Toutefois, la patiente a gardé un érythème rosé couperosique sans infiltration.

## Conclusion

La LC continue à poser un vrai problème de santé publique au Maroc, plus particulièrement, la LC du visage peut se présenter sous différents aspects, ce qui est à l'origine de retard diagnostique. Ainsi, tout dermatologue et otorhinolaryngologiste doit penser à LC devant toute lésion faciale inhabituelle ressemblant à l’érysipèle surtout chez des sujets vivant ou ayant séjourné dans des zones endémique de Leishmanioses.
